# Evaluating the applicability of ivabradine in acute heart failure

**DOI:** 10.1002/clc.24206

**Published:** 2023-12-28

**Authors:** Tzu‐Hsien Tsai, Ming‐Lung Tsai, Dong‐Yi Chen, Yuan Lin, Jian‐Rong Peng, Ning‐I Yang, Ming‐Jui Hung, Tien‐Hsing Chen

**Affiliations:** ^1^ Department of Internal Medicine, Division of Cardiology Ditmanson Medical Foundation Chiayi Christian Hospital Chiayi Taiwan; ^2^ Department of Internal Medicine, Division of Cardiology New Taipei Municipal TuCheng Hospital New Taipei Taiwan; ^3^ College of Medicine Chang Gung University Taoyuan Taiwan; ^4^ College of Management Chang Gung University Taoyuan Taiwan; ^5^ Department of Internal Medicine, Division of Cardiology Linkou Chang Gung Memorial Hospital Taoyuan Taiwan; ^6^ Department of Emergency Medicine Keelung Chang Gung Memorial Hospital Keelung Taiwan; ^7^ Department of Internal Medicine, Division of Cardiology Keelung Chang Gung Memorial Hospital Keelung Taiwan

**Keywords:** acute heart failure, hospitalization, ivabradine

## Abstract

**Background:**

While ivabradine has demonstrated benefits in heart rate control and prognosis for chronic heart failure patients, its application in acute decompensated heart failure remains underexplored.

**Hypothesis:**

For patients with acute decompensated heart failure with reduced ejection fraction (HFrEF) who are intolerant to β‐blockers or unable to further titrate their dosage, the use of ivabradine is hypothesized to be effective and safe is improving outcomes.

**Methods:**

This retrospective, multicenter database analysis included patients with hospitalized decompensated heart failure with a left ventricular ejection fraction of ≤40% from June 1, 2015 to December 31, 2020. The exclusion criteria were a baseline heart rate of <70 bpm, previous use of ivabradine, mortality during admission, existing atrial fibrillation, or atrial flutter. The primary outcome was the composite of cardiovascular death and hospitalization for heart failure.

**Results:**

Of the 4163 HFrEF patients analyzed, 684 (16.4%) were administered ivabradine during their index admission. After matching, there were 617 patients in either group. The results indicated that ivabradine use was not significantly associated with the risk of the primary composite outcome (hazard ratio: 1.10; 95% confidence interval: 0.94−1.29). Similarly, the risk of secondary outcomes and adverse renal events did not significantly differ between the ivabradine and non‐ivabradine cohorts (all *p* > .05).

**Conclusion:**

For hospitalized acute decompensated heart failure patients who are intolerant to β‐blockers or cannot further titrate them, ivabradine offers a consistent therapeutic effect. No significant disparities were noted between the ivabradine and non‐ivabradine groups in heart failure hospitalization and cardiovascular death.

## Graphical abstract

The primary design and outcome of the study.

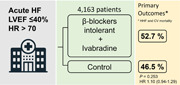

## 1 BACKGROUND

Heart rate stands as an independent risk factor for mortality and cardiovascular events.^1^, ^2^, ^3^ Heart rate reduction can alleviate afterload, relieve left ventricular wall stress, and augment the left ventricle's stroke volume, thereby enhancing heart function and mitigating symptoms of cardiovascular diseases.^4^ While β‐blockers are pivotal for heart failure with reduced ejection fraction (HFrEF),^5^, ^6^ their application is curtailed by side effects such as compromised hemodynamics, diminished cardiac inotropy, and the potential to exacerbate symptoms linked to acute decompensated heart failure.^7^, ^8^

Ivabradine has recently been spotlighted as a potential therapy for HFrEF patients, especially those with sinus tachycardia. It selectively inhibits the pacemaker current in the sinoatrial node, reducing heart rate without affecting myocardial contractility or blood pressure. This unique action stems from its role as a selective inhibitor of the hyperpolarization‐activated cyclic nucleotide‐gated channel in the sinoatrial node, modulating heart rate by decelerating pacemaker cell depolarization.^9^, ^10^ Ivabradine effectively reduces the risk of hospitalization and mortality in patients with chronic HFrEF maintaining sinus rhythm. The SHIFT trial (Systolic Heart Failure Treatment with the I(f) Inhibitor Ivabradine Trial) demonstrated an 18% relative risk reduction in the primary endpoint of cardiovascular death or hospitalization for worsening HF with ivabradine compared with placebo.^11^ Subsequent studies showed significant improvement in the New York Heart Association functional class, quality of life, and left ventricular remodeling with ivabradine.^12^, ^13^ Based on these results, ivabradine has been included in the current guidelines for chronic HFrEF management.^5^, ^6^

According to the health insurance system regulation, ivabradine's use is confined to HFrEF patients who either have contraindications to or exhibit intolerance toward β‐blockers. There's a significant emphasis on patients who are intolerant to β‐blockers. In real‐world scenarios, understanding the outcomes and safety of ivabradine in acute heart failure becomes essential. However, the role of ivabradine for this specific group, particularly those who are either intolerant to β‐blockers or cannot further titrate them, remains a topic of debate. While some studies advocate for its efficacy in acute decompensated HF (ADHF) scenarios, others challenge this notion.^14^, ^15^, ^16^ Given the lack of consensus regarding ivabradine use for ADHF, further clinical research is warranted to evaluate its safety and efficacy.

This study aimed to evaluate the outcomes of ivabradine use in hospitalized patients with ADHF who could not further titrate β‐blockers. By delving into its potential benefits and risks for this specific patient group, we aim to provide clinicians with a nuanced understanding of ivabradine's role in acute heart failure management.

## 2 METHODS

### 2.1 Data source

Data from the Chang Gung Research Database (CGRD), a deidentified database managed by the Chang Gung Memorial Hospital (CGMH) healthcare system, the largest healthcare provider in Taiwan, was used. This system includes four tertiary academic medical centers and three teaching hospitals across Taiwan. The use of data from the CGRD as the basis for accurate estimates in medical studies has been validated.^17^ For data generated before 2015, we used the *International Classification of Diseases*, *Ninth Revision, Clinical Modification* (*ICD*‐*9‐CM*) for diagnosis. Contrarily, for data generated after 2016, we used both the *ICD*‐*9‐CM* and *ICD Tenth Revision* (*ICD‐10‐CM*). More information regarding the CGRD has been published in other articles.^17^, ^18^ Patients' records were anonymized and deidentified before analysis; thus, the requirement for informed consent was waived. This study was approved by the Chang Gung Memorial Hospital Institutional Review Board (202201186B0) and conformed to the principles outlined in the Declaration of Helsinki.^19^

### 2.2 Study group and cohort

**Figure 1:**
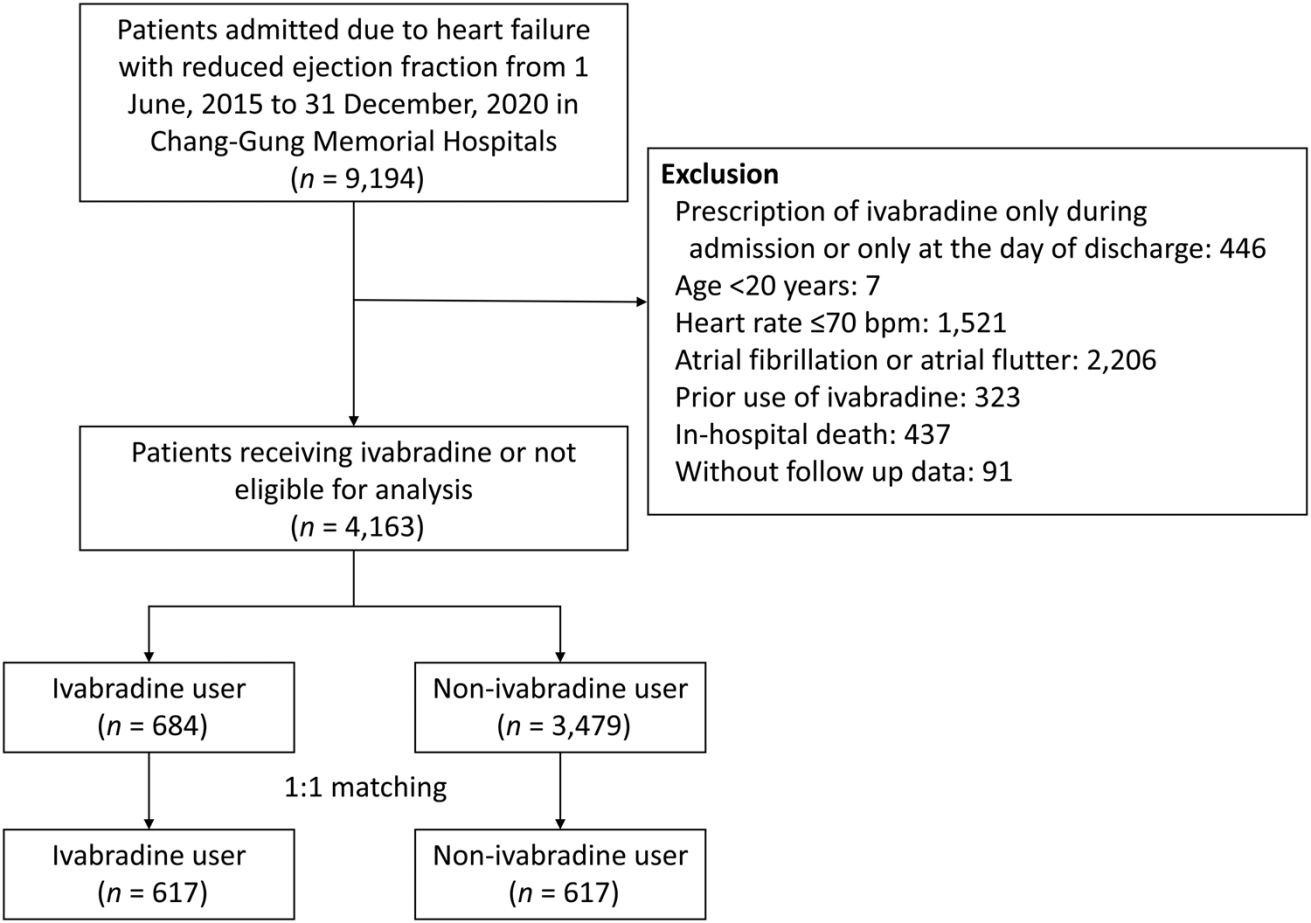
Flowchart of the inclusion and exclusion of the study patients.

The records of patients admitted for decompensated heart failure with left ventricular ejection fraction (LVEF) of ≤40% from June 1, 2015 to December 31, 2020, were obtained from the CGRD. The index date was the date of discharge after the index heart failure admission. Each patient's LVEF was determined based on the echocardiography report generated during the index admission. Patients younger than 20 years, who had a baseline heart rate of ≤70 bpm, previous ivabradine use (before the index admission), a prescription of ivabradine only during admission or only at the day of discharge; a diagnosis of atrial fibrillation (AF) or atrial flutter (AFL) before or during the index admission, and no follow‐up information after the index admission; and who did not survive to discharge were excluded. The remaining patients were divided into two study groups: those who used ivabradine during admission and on discharge (*n* = 684) and those who did not (*n* = 3479) (Figure 1).

### 2.3 Covariate measurements

The covariates of interest were the patients' demographic characteristics (age, sex, smoking, and body mass index [BMI]), baseline vital signs (systolic blood pressure [SBP], diastolic blood pressure, and heart rate, first recorded during the index admission), history of heart failure admission (in the previous year and the prior 3 years), comorbidities (coronary artery disease, diabetes, and nine others), concomitant medications (angiotensin receptor‐neprilysin inhibitors [ARNIs], digoxin, angiotensin‐converting enzyme inhibitors [ACEIs], or angiotensin receptor blockers [ARBs], mineralocorticoid receptor antagonists [MRAs], loop diuretics, and 10 others), laboratory test results (serum creatinine, brain natriuretic peptide, and 18 others), echocardiography results (LVEF, left ventricular end‐diastolic diameter, left ventricular end‐systolic diameter, left atrium diameter, and mitral regurgitation severity), and in‐hospital events (hospital stay, intensive care unit (ICU) stay, shock episodes, intubation, acute coronary syndrome episodes, and percutaneous coronary interventions [PCIs]).

### 2.4 Outcome definitions

The primary outcome was a composite of cardiovascular death or hospitalization for heart failure (HHF) during follow‐up. The secondary outcomes were cardiovascular death, HHF, and all‐cause mortality. HHF was defined as unscheduled hospitalization during which the patient required at least one treatment, including diuretics, nitrites, or inotropes. The dates, places, and causes of patient death were linked to the Taiwan Death Registry database. Cardiovascular death includes death due to acute myocardial infarction, sudden cardiac death, and death due to heart failure, stroke, cardiovascular procedures or hemorrhage, or other cardiovascular causes. Other outcomes included all‐cause admission, newly diagnosed AF or AFL, and myocardial infarction (requiring admission). Renal outcomes comprised worsening renal function (and eGFR decline of >50%), doubling serum creatinine, and renal failure requiring new dialysis during follow‐up. The follow‐up period was defined as the period from the date of index hospitalization to the date of death, outcome occurrence, last visit in CGMHs, or December 31, 2020, whichever occurred first.

### 2.5 Statistical analysis

We created a propensity score‐matched cohort to reduce potential confounding when comparing outcomes between the study groups (ivabradine vs. non‐ivabradine). The propensity score was the predicted probability to be treated with ivabradine given specific covariate values derived from the multivariable logistic regression model. Each patient using ivabradine was matched with one counterpart not using ivabradine. We used the greedy nearest‐neighbor algorithm for matching with a 0.2 caliper, with random matching order, and without replacement. The balance of baseline characteristics between the groups was assessed using the standardized difference (STD), where an absolute STD value of less than 0.1 was considered a negligible difference.

Outcome comparisons between the groups were made in the propensity score‐matched cohort. The risk of fatal outcomes (e.g., composite of cardiovascular death, and HHF, all‐cause death) was compared between groups using the Cox proportional‐hazards model. The cumulative incidence of nonfatal outcomes (e.g., HHF, worsening renal function) between groups was compared using Fine and Gray's subdistribution hazard model, in which death during follow‐up was considered a competing risk. In the survival analyses, the study groups were the only explanatory variable. Subgroup analysis on the primary composite outcome was conducted stratified by the prespecified subgroup variables, including age (<70 vs. ≥70 years), sex, SBP (<90 vs. ≥90 mmHg), cardiogenic shock, PCI, and acute coronary syndrome during the index admission, BMI (<25 vs. ≥25 kg/m^2^), ICU stay, and concomitant use of β‐blocker.

Finally, heart rate data at baseline and 6, 9, and 12 months after discharge were extracted. The heart rate change between the two groups from baseline to follow‐up measurements was compared using a linear mixed model, in which the baseline heart rate (intercept) was treated as a random effect. A two‐sided *p* < .05 was considered statistically significant. All statistical analyses were conducted using SAS version 9.4 (SAS Institute).

## 3 RESULTS

### 3.1 Patient characteristics

After applying the exclusion criteria, 4163 ADHF patients with LVEF ≤40% were finally included in the study. Of them, 684 (16.4%) received ivabradine during the index admission and on the day of discharge (Table 1). Before matching, the ivabradine group had younger patients (61.0 ± 15.8 vs. 64.5 ± 15.5 years), had more smokers, had patients with lower SBP levels and higher baseline heart rate (100.2 ± 17.6 vs. 91.7 ± 15.9 mmHg), and lesser patients with end‐stage renal disease requiring dialysis (absolute STD values >0.1). Furthermore, this group had more patients receiving heart failure medication, including ARNI, ACEI/ARB, MRA, loop diuretics, and digoxin. The crude data in the ivabradine showed lower LVEF (26.1% ± 7.6% vs. 30.5% ± 7.1%) and larger left ventricle end‐systolic dimension (52.4 ± 9.6 vs. 49.4 ± 9.0 mm). In addition, the ivabradine group had poor in‐hospital outcomes, including extended hospitalization, ICU days, respiratory failure requiring intubation, and acute coronary syndrome. After 1:1 matching, no significant difference in the baseline characteristics between the groups was observed (absolute STD values <0.1).

**Table 1:** Baseline characteristics of patients with and without use of ivabradine.

	Before matching				After matching		
Variable	Available number	Ivabradine (*n* = 684)	Non‐ivabradine (*n* = 3479)	STD	Ivabradine (*n* = 617)	Non‐ivabradine (*n* = 617)	STD
Age (year)	4163	61.0 ± 15.8	64.5 ± 15.5	−0.22	61.4 ± 15.7	61.2 ± 15.8	0.02
Male	4163	498 (72.8)	2436 (70.0)	0.06	443 (71.8)	439 (71.2)	0.01
Smoking	4163	308 (45.0)	1221 (35.1)	0.20	266 (43.1)	254 (41.2)	0.04
BMI (kg/m)^2^	4051	25.8 ± 5.3	24.9 ± 4.9	0.16	25.6 ± 5.2	25.5 ± 5.2	0.02
Baseline vital sign							
SBP (mmHg)	4163	126.2 ± 25.7	133.4 ± 26.0	−0.28	127.2 ± 25.7	126.1 ± 24.6	0.04
DBP (mmHg)	4163	80.3 ± 18.2	80.5 ± 18.0	−0.01	80.9 ± 18.1	80.5 ± 19.0	0.02
Heart rate (bpm)	4163	100.2 ± 17.6	91.7 ± 15.9	0.51	99.2 ± 16.9	99.5 ± 20.2	−0.02
HF admission in the previous year	4163	158 (23.1)	621 (17.8)	0.13	137 (22.2)	132 (21.4)	0.02
No of HF admission in the previous 3 years	4163						
0		499 (73.0)	2643 (76.0)	−0.07	456 (73.9)	463 (75.0)	−0.03
1		106 (15.5)	611 (17.6)	−0.06	99 (16.0)	102 (16.5)	−0.01
2		37 (5.4)	120 (3.4)	0.10	31 (5.0)	24 (3.9)	0.05
3−5		32 (4.7)	91 (2.6)	0.11	26 (4.2)	23 (3.7)	0.02
≥6		10 (1.5)	14 (0.4)	0.11	5 (0.8)	5 (0.8)	<0.01
Comorbidity							
Coronary artery disease	4163	351 (51.3)	1718 (49.4)	0.04	307 (49.8)	308 (49.9)	<0.01
Myocardial infarction	4163	77 (11.3)	505 (14.5)	−0.10	69 (11.2)	72 (11.7)	−0.02
Hypertension	4163	432 (63.2)	2507 (72.1)	−0.19	392 (63.5)	390 (63.2)	0.01
Dyslipidemia	4163	289 (42.3)	1666 (47.9)	−0.11	259 (42.0)	263 (42.6)	−0.01
Diabetes mellitus	4163	324 (47.4)	1801 (51.8)	−0.09	296 (48.0)	286 (46.4)	0.03
Chronic kidney disease	4163	278 (40.6)	1703 (49.0)	−0.17	252 (40.8)	258 (41.8)	−0.02
Dialysis	4163	44 (6.4)	424 (12.2)	−0.20	44 (7.1)	40 (6.5)	0.03
Stroke	4163	67 (9.8)	318 (9.1)	0.02	53 (8.6)	53 (8.6)	<0.01
Chronic obstructive pulmonary disease	4163	135 (19.7)	635 (18.3)	0.04	121 (19.6)	119 (19.3)	0.01
Peripheral arterial disease	4163	37 (5.4)	337 (9.7)	−0.16	37 (6.0)	26 (4.2)	0.08
Liver cirrhosis	4163	20 (2.9)	119 (3.4)	−0.03	17 (2.8)	18 (2.9)	−0.01
Medication							
ARNI	4163	106 (15.5)	186 (5.3)	0.34	78 (12.6)	77 (12.5)	<0.01
ACEI/ARB	4163	596 (87.1)	2736 (78.6)	0.23	539 (87.4)	531 (86.1)	0.04
DHP‐CCB	4163	211 (30.8)	1314 (37.8)	−0.15	196 (31.8)	180 (29.2)	0.06
Spironolactone	4163	447 (65.4)	1500 (43.1)	0.46	386 (62.6)	405 (65.6)	−0.06
Eplerenone	4163	16 (2.3)	15 (0.4)	0.16	9 (1.5)	10 (1.6)	−0.01
Loop diuretics	4163	636 (93.0)	2772 (79.7)	0.39	569 (92.2)	572 (92.7)	−0.02
Digoxin	4163	110 (16.1)	308 (8.9)	0.22	86 (13.9)	87 (14.1)	<0.01
Amiodarone	4163	42 (6.1)	182 (5.2)	0.04	35 (5.7)	40 (6.5)	−0.03
SGLT2i	4163	80 (11.7)	215 (6.2)	0.19	67 (10.9)	64 (10.4)	0.02
GLP1RA	4163	3 (0.4)	14 (0.4)	0.01	3 (0.5)	4 (0.6)	−0.02
Other oral hypoglycemic agents	4163	269 (39.3)	1371 (39.4)	<0.01	246 (39.9)	242 (39.2)	0.01
Insulin	4163	242 (35.4)	1088 (31.3)	0.09	216 (35.0)	215 (34.8)	<0.01
Statin	4163	381 (55.7)	1820 (52.3)	0.07	343 (55.6)	335 (54.3)	0.03
Aspirin	4163	493 (72.1)	2339 (67.2)	0.11	442 (71.6)	447 (72.4)	−0.02
P2Y12	4163	387 (56.6)	1852 (53.2)	0.07	346 (56.1)	359 (58.2)	−0.04
Laboratory data							
NT‐pro BNP (pg/mL)	630	2860 [1264−10500]	3867 [1177−16090]	NA	7359 [2306−15075]	6487 [2011−15318]	NA
BNP (pg/mL)	2787	1346 [670−2680]	1394 [652−2761]	NA	1442 [757−2458]	1447 [872−2515]	NA
BUN (mg/dL)	4018	29.7 ± 20.7	33.4 ± 25.4	−0.16	30.0 ± 21.2	29.3 ± 21.4	0.03
Creatinine (mg/dL)	4135	1.8 ± 1.9	2.4 ± 2.7	−0.25	1.9 ± 2.0	1.8 ± 1.9	0.03
eGFR (mL/min/1.73 m)^2^	4135	61.9 ± 31.5	56.4 ± 35.6	0.16	61.6 ± 31.7	61.4 ± 31.3	0.01
Kidney function status	4163						
≥60		375 (54.8)	1581 (45.4)	0.19	334 (54.1)	334 (54.1)	<0.01
30−59		191 (27.9)	896 (25.8)	0.05	173 (28.0)	172 (27.9)	<0.01
<30		74 (10.8)	578 (16.6)	−0.17	66 (10.7)	71 (11.5)	−0.03
Dialysis		44 (6.4)	424 (12.2)	−0.20	44 (7.1)	40 (6.5)	0.03
Sodium (Na) (mEq/L)	4143	137.5 ± 4.3	137.6 ± 4.3	−0.04	137.5 ± 4.3	137.5 ± 4.2	<0.01
Potassium (K) (mEq/L)	4149	4.0 ± 0.6	4.0 ± 0.6	−0.03	4.0 ± 0.6	4.0 ± 0.6	−0.04
Uric acid (mg/dL)	2527	7.7 ± 2.7	7.5 ± 2.6	0.06	7.6 ± 2.3	7.7 ± 2.2	−0.01
AST (U/L)	3092	35.0 [23.0−59.0]	30.0 [22.0−47.0]	NA	35.2 [22.7−59.0]	35.0 [23.3−60.6]	NA
ALT (U/L)	3919	30.0 [18.0−61.0]	24.0 [15.0−42.0]	NA	30.0 [17.0−59.0]	30.0 [18.0−53.0]	NA
LDL‐C (mg/dL)	3340	69.6 ± 47.3	73.8 ± 47.7	−0.09	70.8 ± 44.2	71.5 ± 42.3	−0.02
Total cholesterol (mg/dL)	3323	162.7 ± 44.5	165.0 ± 46.0	−0.05	162.4 ± 40.6	163.9 ± 41.4	−0.04
Hemoglobin (g/dL)	4160	12.9 ± 2.5	12.3 ± 2.6	0.22	12.8 ± 2.5	13.0 ± 2.6	−0.05
HCO_3_	1907	22.4 ± 5.4	22.7 ± 5.0	−0.04	22.8 ± 4.3	22.5 ± 3.9	0.05
Total bilirubin (mg/dL)	2637	1.1 ± 0.9	1.0 ± 0.8	0.13	1.0 ± 0.8	1.0 ± 0.7	0.01
Albumin (mg/dL)	2900	3.5 ± 0.5	3.5 ± 0.6	−0.02	3.6 ± 0.5	3.6 ± 0.5	−0.01
Platelet (count × 10)^3^	4158	234.3 ± 82.8	226.2 ± 86.0	0.10	234.5 ± 81.7	235.2 ± 81.4	−0.01
WBC (count × 10)^3^	4157	10.3 ± 4.5	9.1 ± 3.9	0.28	10.1 ± 4.3	10.0 ± 4.4	0.04
Lactate (mg/dL)	1265	28.1 ± 27.0	24.2 ± 23.9	0.15	24.6 ± 16.9	25.0 ± 17.0	−0.02
INR	3491	1.2 ± 0.2	1.2 ± 0.2	0.05	1.2 ± 0.2	1.2 ± 0.2	0.01
Echocardiography result							
LVEF (%)	4163	26.1 ± 7.6	30.5 ± 7.1	−0.61	26.6 ± 7.4	26.9 ± 7.9	−0.04
LVEDD (mm)	4159	60.5 ± 9.2	59.2 ± 13.3	0.12	60.2 ± 9.1	60.2 ± 9.3	<0.01
LVESD (mm)	4159	52.4 ± 9.6	49.4 ± 9.0	0.32	51.9 ± 9.5	52.0 ± 10.3	<0.01
LA diameter (mm)	4145	43.2 ± 7.8	42.8 ± 7.8	0.05	43.1 ± 7.7	42.9 ± 7.9	0.03
MR severity	4124						
Severe		70 (10.3)	257 (7.5)	0.10	62 (10.0)	67 (10.9)	−0.03
Moderate		172 (25.3)	847 (24.6)	0.02	158 (25.6)	153 (24.8)	0.02
Mild		353 (51.8)	1950 (56.6)	−0.10	315 (51.1)	316 (51.2)	<0.01
Trivial/none		86 (12.6)	389 (11.3)	0.04	79 (12.8)	80 (13.0)	<0.01
In‐hospital event							
Hospital days	4163	15.0 ± 11.6	11.8 ± 10.9	0.29	14.4 ± 11.0	14.1 ± 12.0	0.03
ICU days	4163	3.2 ± 5.5	1.3 ± 3.1	0.42	2.9 ± 5.3	2.7 ± 4.7	0.05
Shock	4163	170 (24.9)	369 (10.6)	0.38	138 (22.4)	141 (22.9)	−0.01
Intubation	4163	38 (5.6)	53 (1.5)	0.22	27 (4.4)	23 (3.7)	0.03
Acute coronary syndrome	4163	181 (26.5)	564 (16.2)	0.25	152 (24.6)	160 (25.9)	−0.03
PCI	4163	151 (22.1)	594 (17.1)	0.13	132 (21.4)	149 (24.1)	−0.07
Follow‐up duration (month)	4163	20.6 ± 16.6	22.5 ± 18.8	−0.11	21.1 ± 16.7	20.6 ± 18.4	0.03

*Note*: Data were presented as frequency (percentage) or mean ± standard deviation or median [25th and 75th percentile].

Abbreviations: ACEI, angiotensin converting enzyme inhibitors; ALT, alanine aminotransferase; ARB, angiotensin receptor blocker; ARNI, angiotensin receptor‐neprilysin inhibitor; AST, aspartate aminotransferase; BMI, body mass index; BNP, brain natriuretic peptide; CCB, calcium‐channel blockers; DBP, diastolic blood pressure; DHP, dihydropyridine; eGFR, estimated glomerular filtration rate; GLP1RA, glucagon‐like peptide 1 receptor agonist; HCO_3_, bicarbonate; HF, heart failure; ICU, intensive care unit; INR, international normalized ratio; LA, left atrial; LDL‐C, low density lipoprotein cholesterol; LVEDD, left ventricular end‐diastolic diameter; LVEF, left ventricular ejection fraction; LVESD, left ventricle end‐systolic dimension; MR, mitral regurgitation; NT‐pro BNP, N terminal pro B type natriuretic peptide; PCI, percutaneous coronary intervention; P2Y12, purinergic receptor P2Y, G‐protein coupled, 12; SBP, systolic blood pressure; SGLT2i, sodium‐glucose cotransporter 2 inhibitors; STD, standardized difference; WBC, white blood cell.

### 3.2 Outcomes

**Figure 2:**
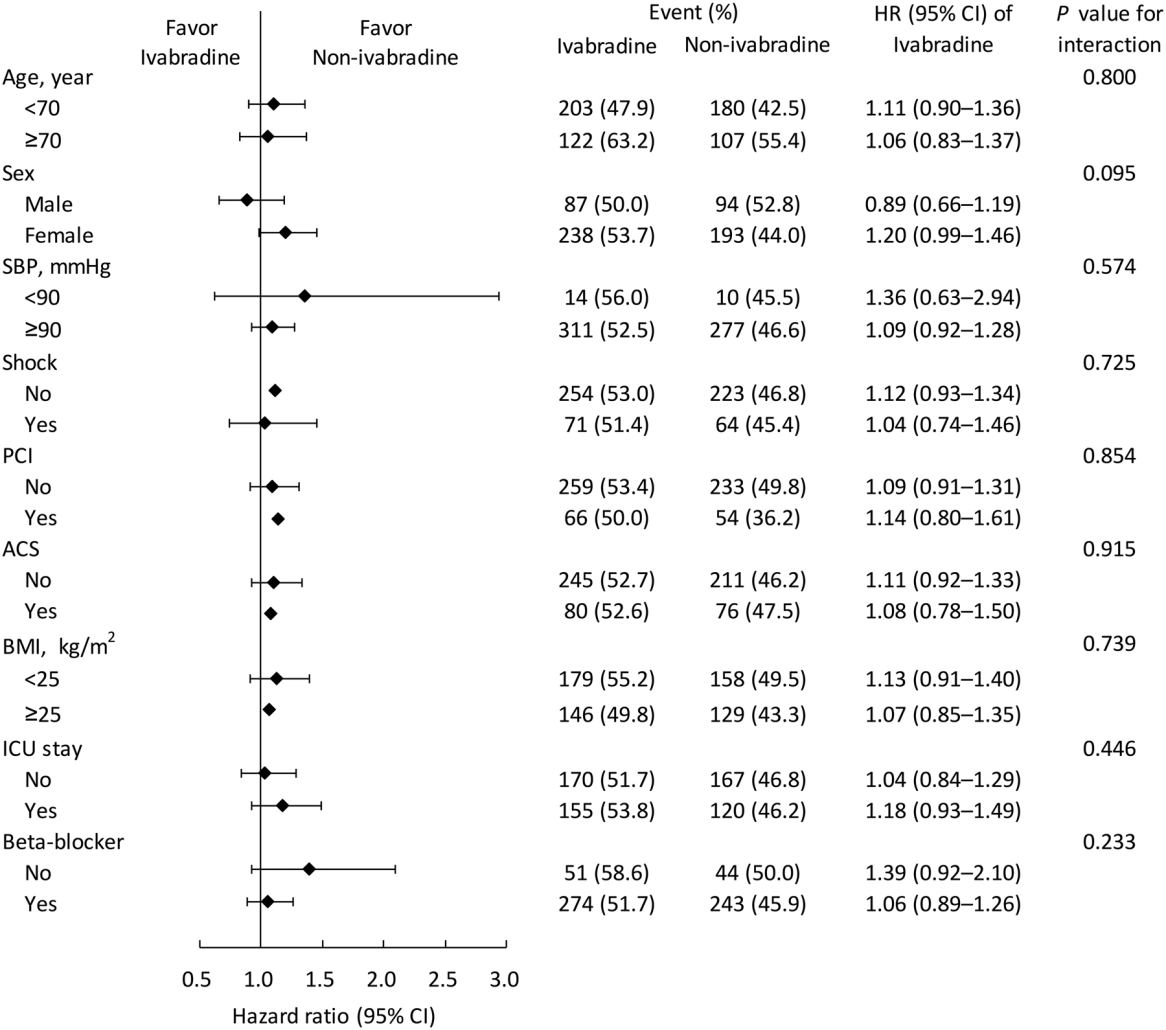
Subgroup analysis on the composite outcome of heart failure hospitalization and cardiovascular death by prespecified subgroup variables in the propensity score‐matched cohort. CI, confidence interval.

In the propensity score‐matched cohort, the mean follow‐up durations were 21.1 ± 16.7 and 20.6 ± 18.4 months for the ivabradine and non‐ivabradine groups, respectively. During follow‐up, 325 (52.7%) and 287 (46.5%) events of the primary composite outcome occurred (Table 2). The results indicated that ivabradine use was not significantly associated with the risk of the primary composite outcome (hazard ratio 1.10: 95% confidence interval: 0.94−1.29; Figure S, 1). Moreover, no significant difference in the risk of secondary outcomes, other outcomes, and adverse renal events was observed between the groups (all *p* values >0.05). The subgroup analysis revealed that the observed neutral effect on the primary composite outcome was consistent with the prespecified subgroups, with nonsignificant interactions (Figure 2 and Supporting Information S3: Table 1). The heart rate changes following discharge were recorded every 3 months (Figure S, 2). The heart rates of both groups had similar reductions during the follow‐up period (*p* for interaction = 0.998).

**Table 2:** Clinical outcomes of patients with and without use of ivabradine in the propensity score matched cohort.

Outcome	Ivabradine (*n* = 617)	Non‐ivabradine (*n* = 617)	HR or SHR (95% CI) of ivabradine	*p*
Primary outcome				
Composite of hospitalization for heart failure and cardiovascular death	325 (52.7)	287 (46.5)	1.10 (0.94−1.29)	.253
Secondary outcome				
All‐cause death	173 (28.0)	145 (23.5)	1.15 (0.92−1.43)	.213
Cardiovascular death	116 (18.8)	89 (14.4)	1.26 (0.96−1.66)	.102
Hospitalization for heart failure	285 (46.2)	247 (40.0)	1.10 (0.93−1.31)	.263
Other outcome				
All‐cause admission	352 (57.1)	311 (50.4)	1.07 (0.92−1.25)	.402
Newly‐diagnosed AF/AFL	30 (4.9)	41 (6.6)	0.68 (0.42−1.09)	.109
Myocardial infarction	34 (5.5)	26 (4.2)	1.25 (0.74−2.10)	.410
Adverse renal event				
Worsening renal function	126 (20.4)	107 (17.3)	1.12 (0.87−1.44)	.394
Creatinine doubling	103 (16.7)	90 (14.6)	1.09 (0.82−1.44)	.550
Dialysis	43 (7.0)	26 (4.2)	1.57 (0.97−2.54)	.068

*Note*: Data is not specified were presented as frequency (percentage).

Abbreviations: AF, atrial fibrillation; AFL, atrial flutter; CI, confidence interval; HR, hazard ratio; SHR, subdistribution hazard ratio.

## 4 DISCUSSION

In real‐world scenarios, ivabradine is often considered for patients who exhibit contraindications to or intolerance of β‐blockers. This study provides insights into the outcomes of such patients. Our study aimed to evaluate the prognosis of ivabradine use in hospitalized patients with acute decompensated HFrEF. While most major trials have predominantly focused on ivabradine's efficacy in treating chronic heart failure, its optimal use in patients with ADHF remains a topic of debate.^20^ We found no significant differences in the composite outcome of HHF and cardiovascular death between the ivabradine and non‐ivabradine groups. Furthermore, there were no significant differences in any secondary outcomes, including mortality, cardiovascular death, admission for cardiovascular events, HHF, newly onset AF, myocardial infarction, or renal outcomes, such as worsening renal function, creatinine doubling, or newly onset dialysis.

In this study, we specifically investigated the outcomes of ivabradine in hospitalized patients with ADHF. Notably, patients who were administered ivabradine were those who could not tolerate higher doses of β‐blockers at the time due to their prevailing conditions. Compared to the non‐ivabradine group, we observed that the group treated with ivabradine consistently demonstrated improved heart rate changes post‐discharge, with no significant difference in their prognosis. Contrary to previous studies that reported improved outcomes with ivabradine in reducing HHF in the acute setting,^16^, ^21^ our findings suggest that in real‐world scenarios, ivabradine provides outcomes consistent with standard treatments. Several factors might explain this discrepancy. First, the primary benefit of ivabradine in heart failure is its ability to control heart rate, thereby ensuring hemodynamic stability. In our cohort, both groups exhibited similar heart rate improvements during follow‐up, which could account for the comparable prognosis post‐acute stage. The health insurance regulations have also positioned ivabradine as a secondary option, leading to its prescription mainly in patients with more advanced conditions. Second, while we anticipated ivabradine to enhance patient mortality and reduce future heart failure readmission rates, our study did not reflect these outcomes. Although ivabradine does improve hemodynamics, its impact on the sympathetic tone is limited. Elevated sympathetic tone, common in ADHF patients, has been linked to adverse heart failure outcomes^22^, ^23^ Unlike β‐blockers, which inhibit sympathetic tone, ivabradine primarily reduces heart rate by inhibiting the funny current in the sinus node.^24^ This distinction might render ivabradine less comprehensively cardioprotective during the acute phase compared to β‐blockers. Additionally, given the retrospective nature of our study, patients weren't randomly assigned to medication choices. As a result, those prescribed ivabradine might have been switched from other treatments due to limited efficacy or intolerance to conventional medications.

Our analysis underscores the pivotal role of ivabradine in clinical settings, particularly for those patients who are intolerant to β‐blockers. In such scenarios, ivabradine emerges as a vital therapeutic option, offering symptom relief and stabilization of hemodynamics, especially during the advanced stages of heart failure. The significance of heart rate control in managing heart failure cannot be overstated, as highlighted by several studies.^25^, ^26^ Past clinical experiences have further reinforced the efficacy of ivabradine, especially for patients grappling with ADHF or cardiogenic shock. These studies have consistently reported the benefits of heart rate reduction and the consequent hemodynamic improvements that ivabradine brings about.^15^, ^27^, ^28^, ^29^ In a notable study by Colombo et al., researchers focused on a cohort of patients with cardiogenic shock who were supported by veno‐arterial extracorporeal membrane oxygenation. Their findings revealed that ivabradine treatment led to a marked reduction in heart rate and a significant enhancement in ventricular stroke volume. Such improvements facilitated a decrease in the need for extracorporeal flow support and reduced vasopressor administration, underscoring the potential of ivabradine in critical care settings.^30^

Our analysis focused on a cohort characterized predominantly by high‐risk ADHF patients, many of whom were contending with significant cardiovascular adversities. During the mean follow‐up duration of 21 months, the data revealed that more than 28% of these patients demised, and upward of 40% necessitated readmission due to heart failure exacerbation. These elevated mortality and readmission rates underscore the intrinsic vulnerability and heightened risk profile of this patient demographic. In such a clinically intricate landscape, the discernment of the nuanced benefits of therapeutic interventions becomes inherently challenging. The pronounced severity of the patients' clinical presentations might overshadow the potential therapeutic efficacies of the interventions, thereby complicating the interpretation of the drug's definitive impact on prognosis. However, an examination of the matched data outcomes offers a nuanced perspective. Both the ivabradine‐treated and non‐ivabradine cohorts exhibited congruent primary and secondary outcomes. Notably, the incidence of adverse events remained consistent across both cohorts, suggesting that the administration of ivabradine did not exacerbate the risk profile of these patients. This observation substantiates the safety profile of ivabradine, particularly when employed for the modulation of heart rate and the optimization of hemodynamics in patients with decompensated heart failure.

## 5 LIMITATION

While our study offers valuable insights into the use of ivabradine in patients with acute decompensated HFrEF, it is not without limitations. First, the inherent nature of our retrospective design introduces potential biases and confounding factors. The absence of random patient enrollment further heightens the risk of selection bias. In clinical practice, ivabradine is often prescribed as an alternative or adjunct to β‐blockers for heart failure patients who cannot tolerate further increases in β‐blocker dosages. Such patients typically find themselves in a more severe or unstable condition when commencing ivabradine. This observation was consistent in our pre‐matching data, where the ivabradine group exhibited signs of more severe conditions, such as lower blood pressure and higher heart rate, and increased usage of heart failure medications. Indicators for the ivabradine group, such as a lower mean LVEF, prolonged hospitalizations, extended ICU stays, increased shock episodes, exacerbated respiratory failure necessitating intubation, and a higher incidence of acute coronary syndromes, further highlight the disparities in baseline characteristics between the groups. Although we employed 1:1 matching to mitigate these differences, some inherent disparities might remain.

Second, our data set lacks specific clinical rationales explaining the inability of certain patients to tolerate β‐blockers. Additionally, we did not have detailed data on the variations and adjustments in β‐blocker dosages across the groups, a significant oversight given that medication dosages often shift in response to a patient's evolving clinical status. During hospitalization for acute heart failure, adjustments to β‐blockers in patients were made based on the clinical judgment of the attending physician, and medication titration, dose reduction, or even discontinuation were performed according to the patient's condition. The 21‐month follow‐up, though considerable, may not fully encapsulate the long‐term outcomes or the sustained effects of ivabradine on acute heart failure patients. Furthermore, the demographics and clinical settings of our study population might differ from those in other studies, potentially affecting the broader applicability of our findings. Given the specific context and design of our study, our findings should be interpreted with caution, especially when comparing them to results from different settings or populations. Nevertheless, our study underscores the importance of further research and the need for more comprehensive evaluations of ivabradine's impact on acute heart failure across diverse patient populations and with extended follow‐up durations.

## 6 CONCLUSION

In conclusion, for hospitalized acute decompensated heart failure patients who are intolerant to β‐blockers or cannot further titrate them, ivabradine offers a consistent therapeutic effect. Our study observed no significant disparities in heart failure hospitalization and cardiovascular death between the ivabradine and non‐ivabradine groups. While the results did not demonstrate a marked improvement in the long‐term prognosis with ivabradine use in this cohort, they did underscore its safety and potential utility in heart rate control and hemodynamic stabilization. Future research, particularly prospective randomized controlled trials or studies focusing on different heart failure populations, will be instrumental in further clarifying the role of ivabradine in heart failure management.

## AUTHOR CONTRIBUTIONS

All authors made substantial, direct, and intellectual contributions to the work and approved its publication. All authors had access to the data outputs and contributed to the writing of the manuscript.

## CONFLICT OF INTEREST STATEMENT

The authors declare no conflict of interest.

## Supporting information

Supporting information.

Supporting information.

Supporting information.

## Data Availability

The original contributions presented in this study are included in the article. Further inquiries can be directed to the corresponding author. The data that support the findings of this study are available on request from the corresponding author. The data are not publicly available due to privacy or ethical restrictions.
