# Supplementary material for: Evaluating the applicability of ivabradine in acute heart failure
Source: Clin Cardiol. 2023 Dec 28;47(1):e24206. doi: 10.1002/clc.24206 (PMC10765997; doi:10.1002/clc.24206)
Supplement: Supplementary file 1 — Supporting information. [file CLC-47-e24206-s003.docx]

**Supplementary Table 1.** Subgroup of the composite of heart failure hospitalization and cardiovascular death outcome.

| Subgroup | Ivabradine  (*n* = 617) | Non-ivabradine  (*n* = 617) | HR (95% CI) of Ivabradine | *P* for interaction |
| --- | --- | --- | --- | --- |
| Age |  |  |  | 0.800 |
| <70 years | 203 (47.9) | 180 (42.5) | 1.11 (0.90–1.36) |  |
| ≥70 years | 122 (63.2) | 107 (55.4) | 1.06 (0.83–1.37) |  |
| Sex |  |  |  | 0.095 |
| Male | 87 (50.0) | 94 (52.8) | 0.89 (0.66–1.19) |  |
| Female | 238 (53.7) | 193 (44.0) | 1.20 (0.99–1.46) |  |
| SBP |  |  |  | 0.574 |
| <90 mmHg | 14 (56.0) | 10 (45.5) | 1.36 (0.63–2.94) |  |
| ≥90 mmHg | 311 (52.5) | 277 (46.6) | 1.09 (0.92–1.28) |  |
| Shock |  |  |  | 0.725 |
| No | 254 (53.0) | 223 (46.8) | 1.12 (0.93–1.34) |  |
| Yes | 71 (51.4) | 64 (45.4) | 1.04 (0.74–1.46) |  |
| PCI |  |  |  | 0.854 |
| No | 259 (53.4) | 233 (49.8) | 1.09 (0.91–1.31) |  |
| Yes | 66 (50.0) | 54 (36.2) | 1.14 (0.80–1.61) |  |
| ACS |  |  |  | 0.915 |
| No | 245 (52.7) | 211 (46.2) | 1.11 (0.92–1.33) |  |
| Yes | 80 (52.6) | 76 (47.5) | 1.08 (0.78–1.50) |  |
| BMI |  |  |  | 0.739 |
| <25 kg/m^2^ | 179 (55.2) | 158 (49.5) | 1.13 (0.91–1.40) |  |
| ≥25 kg/m^2^ | 146 (49.8) | 129 (43.3) | 1.07 (0.85–1.35) |  |
| ICU day |  |  |  | 0.446 |
| <1 day | 170 (51.7) | 167 (46.8) | 1.04 (0.84–1.29) |  |
| ≥1 day | 155 (53.8) | 120 (46.2) | 1.18 (0.93–1.49) |  |
| Beta-blocker |  |  |  | 0.233 |
| No | 51 (58.6) | 44 (50.0) | 1.39 (0.92–2.10) |  |
| Yes | 274 (51.7) | 243 (45.9) | 1.06 (0.89–1.26) |  |

Abbreviations: HR, hazard ratio; CI, confidence interval;

Data were presented as frequency (percentage);

**Supplementary Figure 1.** Cumulative event rate of the composite outcome of heart failure hospitalization and cardiovascular death for ivabradine and non-ivabradine users in the propensity score-matched cohort.

**Supplementary Figure 2.** Long-term heart rate changes of ivabradine and non-ivabradine users in the propensity score-matched cohort.
