# Supplementary figures and images for: Evaluating the applicability of ivabradine in acute heart failure
Source: Clin Cardiol. 2023 Dec 28;47(1):e24206. doi: 10.1002/clc.24206 (PMC10765997; doi:10.1002/clc.24206)

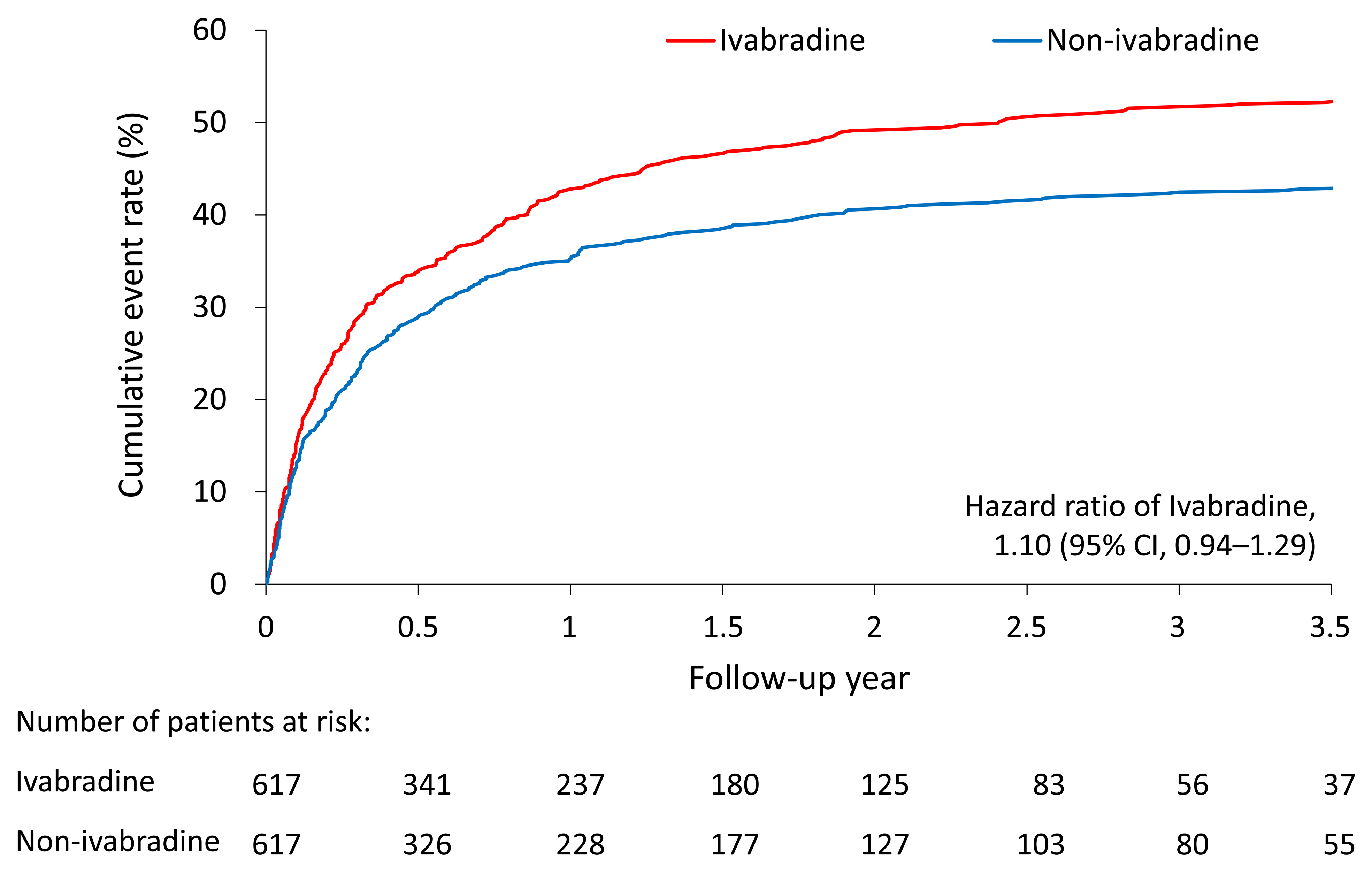

Supplement: Supplementary file 2 — Supporting information. [file CLC-47-e24206-s002.tif]

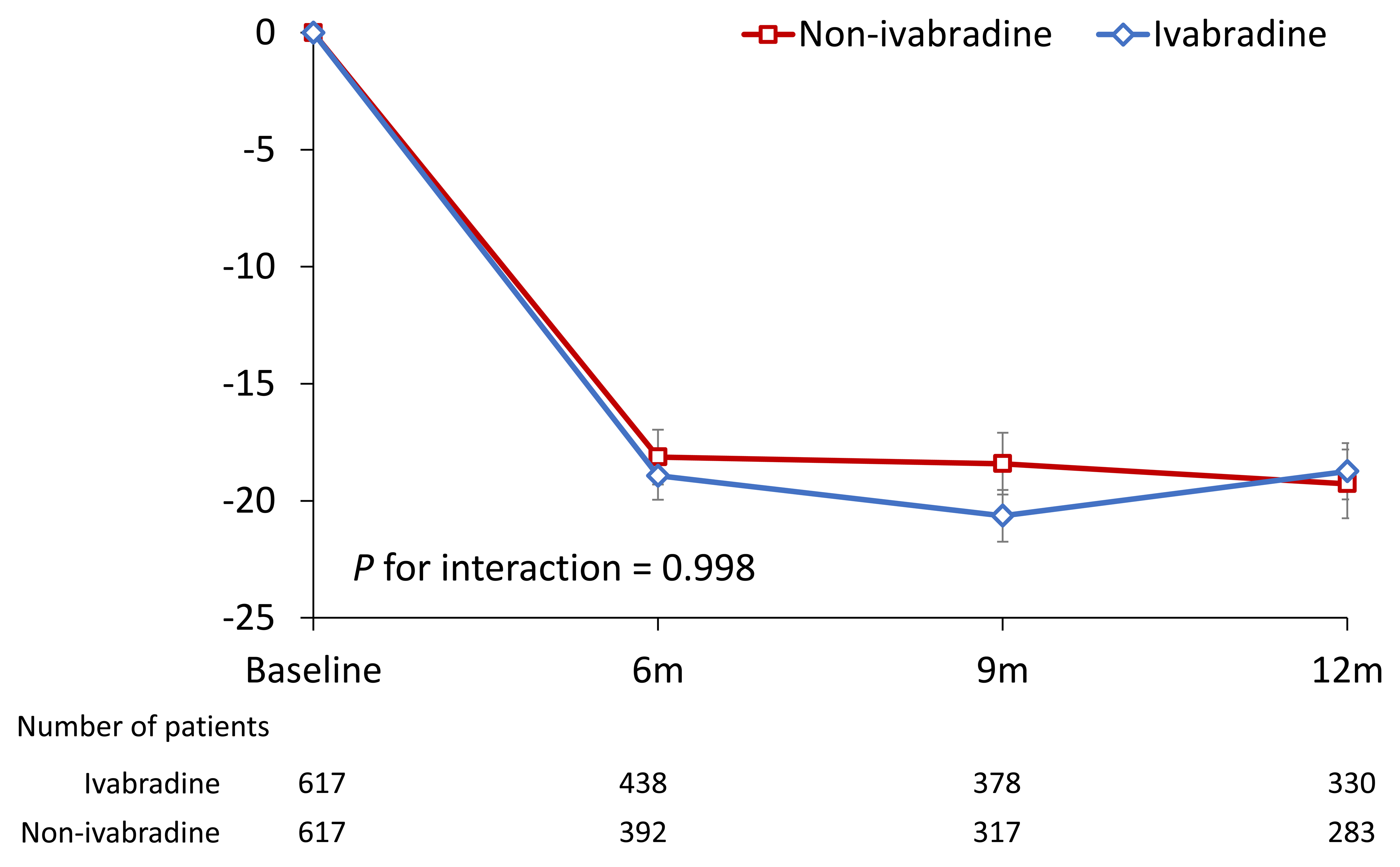

Supplement: Supplementary file 3 — Supporting information. [file CLC-47-e24206-s001.tif]
